# Guillain Barré Syndrome Mimicking Brain Death: A Case Report

**DOI:** 10.7759/cureus.76297

**Published:** 2024-12-24

**Authors:** Lijo James, Aiswarya Mohan, Jithu Mol Thankam, Jeeva George

**Affiliations:** 1 Neurology, Caritas Hospital, Kottayam, IND; 2 Research and Development Cell, Caritas Hospital and Institute of Health Sciences, Kottayam, IND; 3 Neurocritical Care, Caritas Hospital and Institute of Health Sciences, Kottayam, IND

**Keywords:** false brain death, guillain-barré syndrome (gbs), immunotherapy, quadriparesis, respiratory failure

## Abstract

Here, we present a case of Guillain-Barré syndrome (GBS) that mimicked brain death. A 66-year-old lady with a medical history of breast cancer (now receiving hormone therapy), hypertension, and hypothyroidism, presented to the emergency department. The patient was admitted to the neuro ICU with absent brainstem and spinal cord responses, concerning for possible brain death. Further evaluation, however, identified the uncommon GBS with respiratory failure. This case emphasizes the need to recognize this severe manifestation of GBS since misinterpreting this as brain death might result in the discontinuation of ventilatory support.

## Introduction

Guillain-Barré syndrome (GBS) is a category of acute, immune-mediated polyneuropathies that are clinically distinguished through rapidly increasing, symmetrical, ascending weakness and hyporeflexia [1]. The two most common forms of GBS are acute inflammatory demyelinating polyneuropathy (AIDP) and acute motor axonal neuropathy, with AIDP contributing to 60-80% of cases [2,3]. GBS commonly presents with progressive weakness and sensory changes. Its manifestations can vary significantly, making early diagnosis challenging, especially in atypical or extreme cases. One such rare presentation is GBS mimicking false brain stem death, a condition that requires urgent attention and appropriate management to prevent potentially fatal outcomes. GBS can present with such profound neurological and neuromuscular decline that it may be mistaken for brain stem death. GBS presenting with features suggestive of brain death is an exceedingly rare occurrence, with only a limited number of cases reported in the literature [3-5]. The pathophysiology of GBS is thought to be initiated by a cross-reactive immune response. GBS could occur at any age. About 25% of patients develop respiratory insufficiency and show signs of autonomic dysfunction [6]. Here, we discuss a case that shows the importance of recognizing GBS as a potential cause of severe coma that can mimic absent brain death and respiratory failure.

## Case presentation

A 66-year-old woman with a medical history, including breast cancer (currently undergoing immunotherapy), hypertension, and hypothyroidism, presented with progressive neurological symptoms. She experienced paresthesia (tingling or numbness) and progressive weakness in all four limbs for one day. She showed features of autonomic dysfunction in the form of BP (160/90 mmHg) fluctuation and sweating. On clinical examination, she was conscious and oriented, but significant weakness was noted in her neck flexors and limbs, with strength graded 4/5 in the upper limbs and 3/5 in the lower limbs. Her condition rapidly deteriorated, and by the second day, she developed severe respiratory distress, necessitating mechanical ventilation. Over a few days, she became completely comatose with dilated pupils and absent gag reflex, indicating a loss of brainstem reflexes. A caloric test was done, which showed a positive test indicating brain stem inactivity, along with autonomic dysfunction and absent brainstem reflexes.

**Figure 1:**
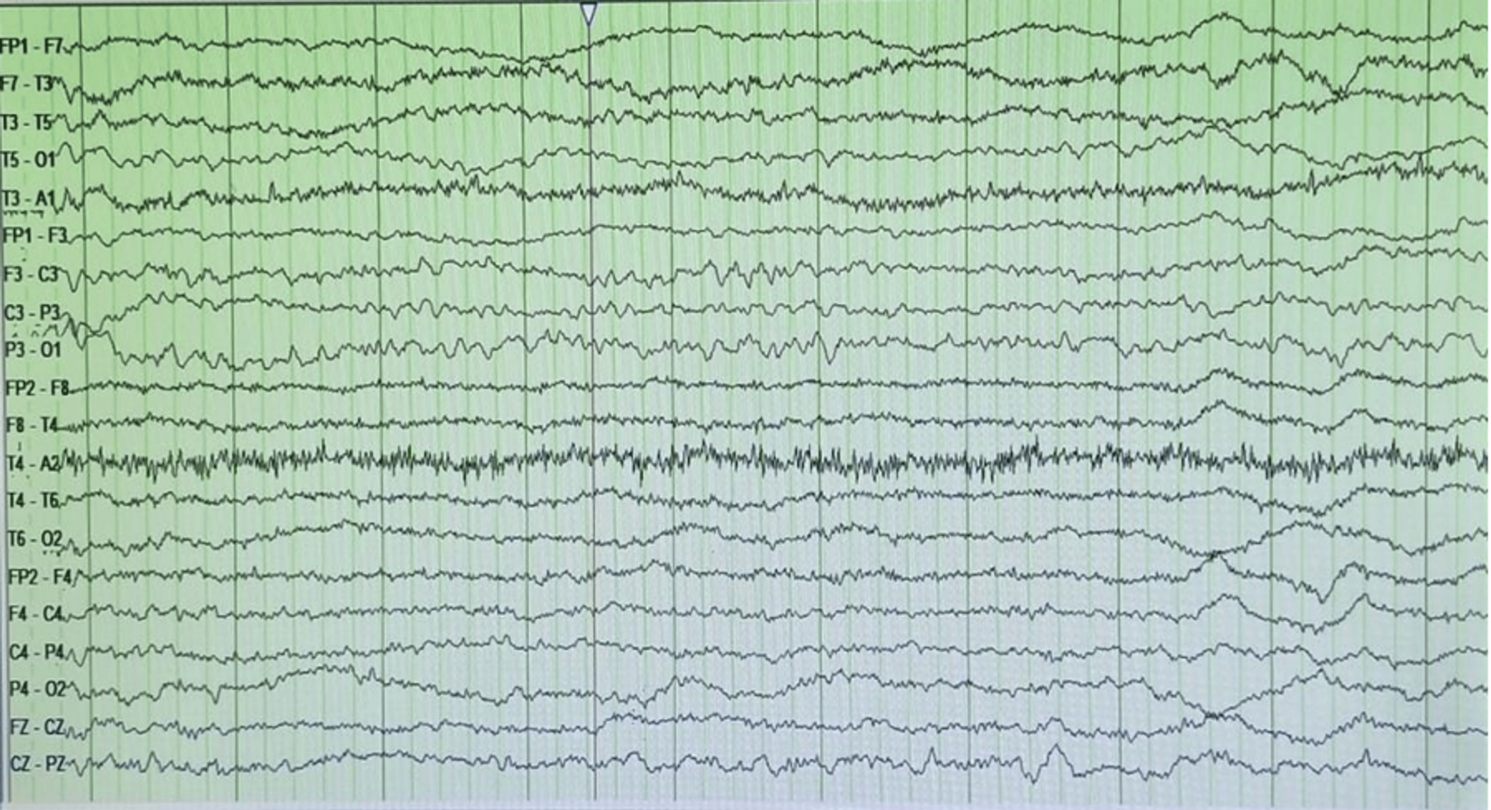
EEG showing normal cortical activity The electroencephalogram (EEG) recording demonstrates normal cortical activity with a consistent and symmetrical background rhythm. The posterior dominant rhythm (PDR) appears intact. There are no signs of epileptiform activity, sharp waves, or generalized slowing, indicating the absence of pathological findings. Additionally, this segment does not display sleep-related features such as sleep spindles (12–14 Hz bursts), K-complexes (high-amplitude sharp transients), or vertex waves, which are hallmarks of Stage 2 sleep. This suggests an awake state. EEG findings are consistent with normal cortical function, without evidence of focal or generalized abnormalities.

An initial investigation, including an MRI brain was done, which was unremarkable, and an MRI of the spine indicated normal spinal cord signal intensity without any intrinsic or extrinsic lesions. Although a nerve conduction study (NCS) could have aided in confirming the diagnosis, it was not conducted as it was not technically feasible at that time. An initial CSF analysis was done on day 3 and results were normal, with no significant abnormalities. Given her history of malignancy, a paraneoplastic neurological profile was tested, but the results were negative. An EEG was conducted, and the results were normal, suggesting normal cortical activity (Figure 1).

A second CSF analysis was done due to the persistence of symptoms; it confirmed elevated protein levels, further supporting the diagnosis of GBS (Table 1).

**Table 1:** Cerebrospinal fluid (CSF) analysis with reference ranges

Test Parameter	1^st^ CSF	2^nd^ CSF 2	Reference Range
CSF TC (Total Cells)	3	1	0-5 cells/mm³
CSF DC (Differential Count - Poly)	Nil	Nil	0%
CSF Lymphocytes	Occasional	Occasional	60-70% of total cells
CSF RBC (Red Blood Cells)	1-2 /HPF	4-6 /HPF	0 cells/mm³
CSF Sugar (Glucose)	99 mg/dL	78 mg/dL	45-80 mg/dL (depends on blood glucose)
CSF Protein	42 mg/dL	251 mg/dL	15-45 mg/dL
Corresponding Blood Sugar	160 mg/dL	141 mg/dL	60-100 mg/dL (fasting)

Suspicion of GBS arose following the rapid progression of her neurological symptoms, particularly after the CSF analysis revealed albuminocytologic dissociation consistent with GBS elevated protein levels. After one week in a coma and completion of intravenous immunoglobulin (IVIG) therapy. Her pupils began to react and her brain stem reflexes gradually returned to normal.

## Discussion

GBS, first described in 1916, is an autoimmune-related acute neuropathy that typically manifests as symmetric ascending flaccid weakness of the lower limbs that can spread to the upper limbs, diaphragm, and cranial nerves over hours to days, with or without sensory and autonomic symptoms [7,8]. The weakening of the diaphragm can lead to respiratory impairment. Our patient suffered from GBS mimicking brainstem death.

GBS has an incidence of about 1 to 2 per 100,000 people per year and can affect individuals of any age, though it is most common in adults [9,10]. Our case presents a unique challenge showing GBS mimicking brain death, which is a rare condition with very few cases documented in the literature [11-13].

In our case, the patient was on ventilator support due to respiratory distress. Approximately 20% of GBS cases result in respiratory failure due to diaphragm paralysis, which can necessitate mechanical ventilation [14,15]. Our patient developed GBS and had a clinical exam resembling brain death. There should be an underlying cause of brain death, and any reversible factors should be eliminated first. Our patient's EEG also indicated normal cortical activity. Cortical functioning and imaging did not show any irreversible damage, ruling out brain death [9,12,16]. Another case report discusses a patient with severe GBS who presented with signs of brain death such as quadriplegia and lack of brainstem reflexes. It underscores the difficulty in differentiating GBS from brain death, which is relevant to the current case [8].

In our case, an electroencephalogram (EEG) ruled out the initial suspicion of brain death. These tests were critical in distinguishing actual brain death from the severe, though potentially reversible, neurological decline found in GBS [12,17,18]. The suspicion of GBS was confirmed by a repeated CSF analysis, which is an important diagnostic tool. Albuminocytologic dissociation is a key finding in CSF. This dissociation occurs because GBS is a demyelinating disease of the peripheral nerves, which causes leakage of proteins from inflamed or damaged nerves into the CSF, without involving an infectious or inflammatory process that would increase white cells. In our case the patient was on immunotherapy, which is thought to overactivate the immune system, resulting in a chain reaction of autoimmune responses. Some literature has reported GBS following therapy with immune checkpoint inhibitors (ICIs), particularly in cancer patients. ICIs are newer options of therapy in cancer patients and have helped achieve success in survival in different cancers. Another study analyzed GBS associated with ICI and found that GBS with potentially life-threatening consequences occurred in 0.1-0.2% of patients treated with ICIs [16]. Our case also contributes to this evolving body of evidence by demonstrating the rapid neurological decline associated with GBS in cancer patients receiving ICIs [19].

In our case, the patient's history of breast cancer and ongoing immunotherapy may have contributed to the initial development of GBS in this case. The management with IVIG therapy sped up the recovery [19].

## Conclusions

This case highlights the importance of recognizing GBS as a potential cause of severe coma that can mimic brain death and respiratory distress. Further diagnostic evaluation, including normal brain activity on EEG and CSF results with albuminocytological dissociation, pointed toward a rare, severe form of GBS. Early identification and initiation of IVIG therapy was a crucial step in the management.

Thorough history-taking with repeated investigations was crucial in accurately diagnosing GBS. This case underscores the significance of conducting a comprehensive neurological exam in patients with atypical presentations to ensure an accurate diagnosis and avoid potentially fatal misdiagnosis. When patients appear atypically, the diagnosis of GBS is commonly missed. We report this case to raise awareness of the possibility that GBS may initially emerge with symptoms resembling false brain stem death.
